# 5-Amino-1*H*-pyrazol-2-ium hydrogen succinate

**DOI:** 10.1107/S1600536814001615

**Published:** 2014-01-29

**Authors:** Thammarse S. Yamuna, Manpreet Kaur, Brian J. Anderson, Jerry P. Jasinski, H. S. Yathirajan

**Affiliations:** a Department of Studies in Chemistry, University of Mysore, Manasagangotri, Mysore 570 006, India; b Department of Chemistry, Keene State College, 229 Main Street, Keene, NH 03435-2001, USA

## Abstract

In the cation of the title salt, C_3_H_6_N_3_^+^·C_4_H_5_O_4_^−^, the protonated pyrazolium ring is planar (r.m.s. deviation = 0.012 Å). An intra­molecular C—H⋯O hydrogen bond occurs in the anion. In the crystal, N—H⋯O hydrogen bonds and a weak C—H⋯O inter­action between the cations and anions form two sets of *R*_2_^2^(8) graph-set ring motifs. Inter­molecular O—H⋯O hydrogen bonds between these lead to a criss-cross pattern along the *b* axis. In addition to the classical hydrogen bonds, a weak C—H⋯π(pyrazolium) inter­action is observed and contributes to crystal packing. All of these inter­actions link the mol­ecules into a two-dimensional supra­molecular framework parallel to (10-1).

## Related literature   

For the broad spectrum of biological properties of pyrazoles, see: Hall *et al.* (2009 ▶) and for their biological and medicinal activities, see: Vinogradov *et al.* (1994 ▶). For succinic acid derivatives used in chemicals, food and pharmaceuticals, see: Sauer *et al.* (2008 ▶). For related structures, see: Kavitha *et al.* (2013 ▶); Kettmann *et al.* (2005 ▶); Koziol *et al.* (2006 ▶); Parvez *et al.* (2001 ▶); Yamuna *et al.* (2013 ▶).
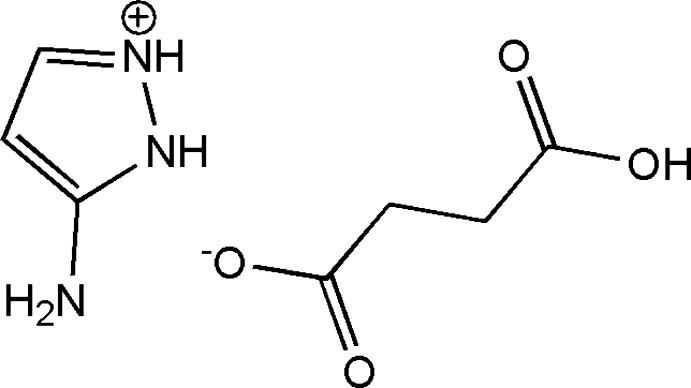


## Experimental   

### 

#### Crystal data  

C_3_H_6_N_3_^+^·C_4_H_5_O_4_^−^
*M* *_r_* = 201.19
Monoclinic, 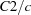

*a* = 18.525 (3) Å
*b* = 6.7872 (9) Å
*c* = 14.564 (3) Å
β = 108.900 (18)°
*V* = 1732.4 (5) Å^3^
*Z* = 8
Mo *K*α radiation
μ = 0.13 mm^−1^
*T* = 173 K
0.32 × 0.24 × 0.12 mm

#### Data collection  

Agilent Eos Gemini diffractometer
Absorption correction: multi-scan (*CrysAlis PRO* and *CrysAlis RED*; Agilent, 2012 ▶) *T*_min_ = 0.591, *T*_max_ = 1.000
5745 measured reflections
2922 independent reflections
1925 reflections with *I* > 2σ(*I*)
*R*_int_ = 0.053

#### Refinement  

*R*[*F* ^2^ > 2σ(*F* ^2^)] = 0.071
*wR*(*F* ^2^) = 0.212
*S* = 1.08
2922 reflections
136 parameters
H atoms treated by a mixture of independent and constrained refinement
Δρ_max_ = 0.36 e Å^−3^
Δρ_min_ = −0.40 e Å^−3^

### 

Data collection: *CrysAlis PRO* (Agilent, 2012 ▶); cell refinement: *CrysAlis PRO*; data reduction: *CrysAlis RED* (Agilent, 2012 ▶); program(s) used to solve structure: *SUPERFLIP* (Palatinus & Chapuis, 2007 ▶); program(s) used to refine structure: *SHELXL2012* (Sheldrick, 2008 ▶); molecular graphics: *OLEX2* (Dolomanov *et al.*, 2009 ▶); software used to prepare material for publication: *OLEX2*.

## Supplementary Material

Crystal structure: contains datablock(s) I. DOI: 10.1107/S1600536814001615/tk5290sup1.cif

Structure factors: contains datablock(s) I. DOI: 10.1107/S1600536814001615/tk5290Isup2.hkl

Supporting information file. DOI: 10.1107/S1600536814001615/tk5290Isup3.cml

CCDC reference:

Additional supporting information:  crystallographic information; 3D view; checkCIF report

## Figures and Tables

**Table 1:** Hydrogen-bond geometry (Å, °) *Cg*1 is the centroid of the pyrazolium ring.

*D*—H⋯*A*	*D*—H	H⋯*A*	*D*⋯*A*	*D*—H⋯*A*
O1*B*—H1*B*⋯O3*B* ^i^	0.82	1.79	2.5832 (18)	164
N1*A*—H1*AA*⋯O4*B* ^ii^	0.86	2.08	2.874 (2)	153
N1*A*—H1*AB*⋯O2*B* ^iii^	0.86	2.07	2.923 (2)	170
N2*A*—H2*A*⋯O3*B* ^ii^	0.95 (3)	1.76 (3)	2.7132 (19)	174 (3)
N3*A*—H3*A*⋯O4*B* ^iv^	0.94 (3)	1.79 (3)	2.672 (2)	156 (3)
C2*A*—H2*AA*⋯O1*B* ^iii^	0.93	2.54	3.372 (2)	148
C3*A*—H3*AA*⋯O2*B*	0.93	2.47	3.214 (2)	138
C3*B*—H3*BA*⋯*Cg*1^v^	0.97	2.69	3.511 (2)	142

Symmetry codes: (i) 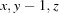; (ii) 
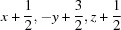
; (iii) 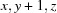; (iv) 
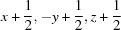
; (v) 
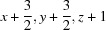
.

**Fig. 1.**
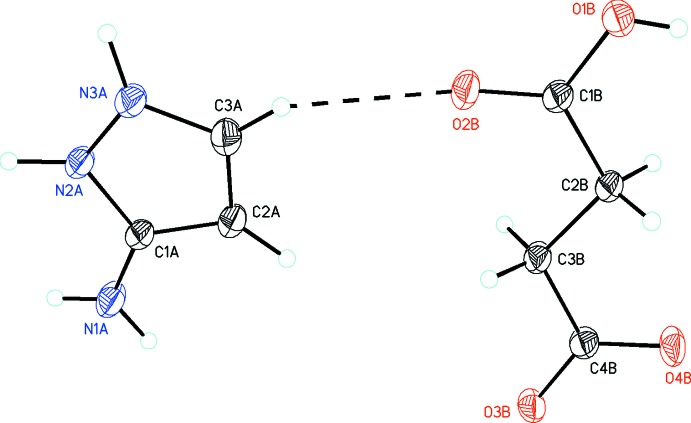
ORTEP drawing of (I) (C3H6N3+.C4H5O4-) showing the labeling scheme with 30% probability displacement ellipsoids. Dashed lines indicate a C3A—H3AA···O1B intermolecular hydrogen bond linking the cation and anion within the asymmetric unit.

**Fig. 2.**
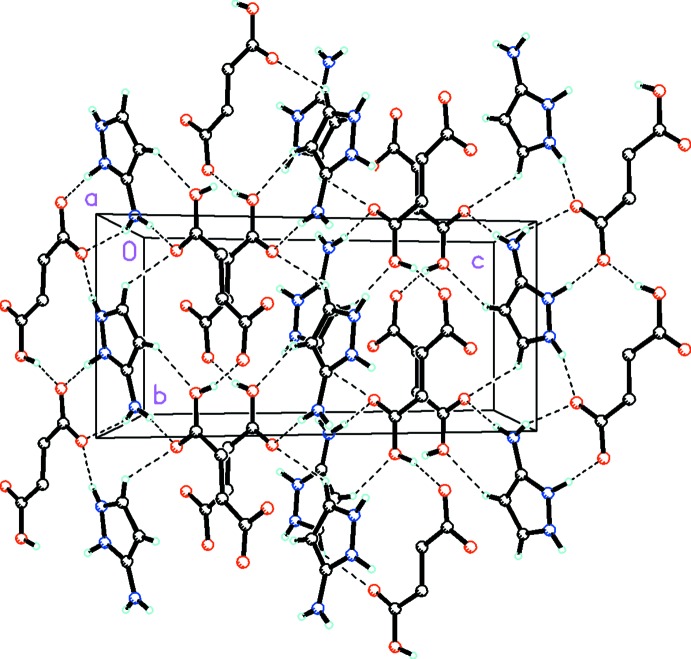
Molecular packing for (I) viewed along the a axis. Dashed lines indicate O—H···O, N—H···O hydrogen bonds and weak C—H···O intermolecular interactions forming R22(8) graph set ring motifs. H atoms not involved in hydrogen bonding have been removed for clarity.

## supplementary crystallographic information

### 2.1. Synthesis and crystallization

A mixture of commercially available 3-amino­pyrazole (0.5 g, 6.02 mmol) and succinic acid (0.71 g, 6.02 mmol) were dissolved in 5 ml of hot di­methyl­sulfoxide. The reaction mixture was stirred for 15 mins at 323 K. The resulting solution was allowed to cool slowly at room temperature upon which X-ray quality crystals of the title salt were obtained after few days; M.pt: 368–373 K.

### 2.2. Refinement

The N-bound H2A and H3A atoms were located by a difference map and refined isotropically. All of the remaining H atoms were placed in their calculated positions and then refined using the riding model with atom—H lengths of 0.93Å (CH); 0.97Å (CH_2_); 0.82Å (OH) or 0.86Å (NH). Isotropic displacement parameters for these atoms were set to 1.2 (CH, CH_2_, NH) or 1.5 (OH) x *U*_eq_ of the parent atom.

### 3. Results and discussion

Pyrazoles comprise an important class of heterocyclic compounds and many pyrazole derivatives are reported to have a broad spectrum of biological properties, such as anti-bacterial and anti-inflammatory activities, anti-cancer (Hall *et al.*, 2009). The chemistry of amino­pyrazoles has been extensively investigated in the past. The considerable biological and medicinal activities of pyrazoles (Vinogradov *et al.*, 1994), for which amino­pyrazoles are preferred precursors, have stimulated these investigations. Succinic acid derivatives are also mostly being used in chemicals, food and pharmaceuticals (Sauer *et al.*, 2008). Recently, the crystal structure of 3-amino­pyrazolium tri­fluoro­acetate (Yamuna *et al.*, 2013) was reported from our research group. Some other structures of related compounds, *viz*., 4-[bis­(4-fluoro­phenyl) methyl]-1-[(2*E*)-3-phenyl­prop-2-en-1-yl]piperazin-1-ium 3-carb­oxy­propano­ate (Kavitha *et al.*, 2013), doxyl­amine hydrogen succinate (Parvez *et al.*, 2001), 5-amino-4-methyl­sulfonyl-1-phenyl-1*H*-pyrazole (Kettmann *et al.*, 2005) and ethyl 1-acetyl-3-amino-1*H*-pyrazole-4-carboxyl­ate (Koziol *et al.*, 2006) have also been reported. In continuation of our work on pyrazoles, this paper reports the crystal structure of the title salt, 5-amino-1*H*-pyrazol-2-ium hydrogen succinate, C_3_H_6_N_3_^+^.C_4_H_5_O_4_^-^, (I).

The title salt, (I), crystallizes with one independent monocation (A) and a monoanion (B) in the asymmetric unit (Fig. 1). In the cation the protonated pyrazolium ring is planar. In the crystal, N—H···O hydrogen bonds involving two hydrogen atoms on the amino group (H1AA, H1AB), a N2A—H2A···O3B inter­molecular hydrogen bond and a weak C2A—H2AA···O1B inter­molecular inter­action between cations and anions form two sets of R_2_^2^(8) graph set ring motifs (Fig. 2). Inter­molecular O—H···O hydrogen bonds between the anions leads to a criss-cross pattern along the *b* axis. In addition to the classical hydrogen bonds, a weak C—H···Cg(pyrazolium) inter­molecular inter­action is observed and contributes to crystal packing. All of these inter­actions directly link the molecules into a 2D supra­molecular framework along (1 0 -1).

### Crystal data

**Table:**

C_3_H_6_N_3_^+^·C_4_H_5_O_4_^−^	*F*(000) = 848
*M**_r_* = 201.19	*D*_x_ = 1.543 Mg m^−^^3^
Monoclinic, *C*2/*c*	Mo *K*α radiation, λ = 0.71073 Å
*a* = 18.525 (3) Å	Cell parameters from 1155 reflections
*b* = 6.7872 (9) Å	θ = 3.2–32.9°
*c* = 14.564 (3) Å	µ = 0.13 mm^−^^1^
β = 108.900 (18)°	*T* = 173 K
*V* = 1732.4 (5) Å^3^	Irregular, colorless
*Z* = 8	0.32 × 0.24 × 0.12 mm

### Data collection

**Table:**

Agilent Eos Gemini diffractometer	2922 independent reflections
Radiation source: Enhance (Mo) X-ray Source	1925 reflections with *I* > 2σ(*I*)
Detector resolution: 16.0416 pixels mm^-1^	*R*_int_ = 0.053
ω scans	θ_max_ = 33.0°, θ_min_ = 3.2°
Absorption correction: multi-scan (*CrysAlis PRO* and *CrysAlis RED*; Agilent, 2012)	*h* = −11→27
*T*_min_ = 0.591, *T*_max_ = 1.000	*k* = −9→9
5745 measured reflections	*l* = −22→20

### Refinement

**Table:**

Refinement on *F*^2^	Primary atom site location: structure-invariant direct methods
Least-squares matrix: full	Hydrogen site location: mixed
*R*[*F*^2^ > 2σ(*F*^2^)] = 0.071	H atoms treated by a mixture of independent and constrained refinement
*wR*(*F*^2^) = 0.212	*w* = 1/[σ^2^(*F*_o_^2^) + (0.1004*P*)^2^] where *P* = (*F*_o_^2^ + 2*F*_c_^2^)/3
*S* = 1.08	(Δ/σ)_max_ < 0.001
2922 reflections	Δρ_max_ = 0.36 e Å^−^^3^
136 parameters	Δρ_min_ = −0.40 e Å^−^^3^
0 restraints	

### Special details

**Table:**

Geometry. All esds (except the esd in the dihedral angle between two l.s. planes) are estimated using the full covariance matrix. The cell esds are taken into account individually in the estimation of esds in distances, angles and torsion angles; correlations between esds in cell parameters are only used when they are defined by crystal symmetry. An approximate (isotropic) treatment of cell esds is used for estimating esds involving l.s. planes.

### Fractional atomic coordinates and isotropic or equivalent isotropic displacement parameters (Å^2^)

**Table:**

	*x*	*y*	*z*	*U*_iso_*/*U*_eq_	
O1B	0.43675 (7)	−0.1627 (2)	0.31692 (11)	0.0344 (4)	
H1B	0.3996	−0.1991	0.2719	0.052*	
O2B	0.49764 (7)	0.1136 (2)	0.36893 (11)	0.0333 (4)	
O3B	0.32006 (7)	0.6651 (2)	0.19603 (10)	0.0272 (3)	
O4B	0.26029 (7)	0.3890 (2)	0.13537 (12)	0.0363 (4)	
C1B	0.44071 (9)	0.0315 (3)	0.31702 (12)	0.0229 (4)	
C2B	0.37281 (9)	0.1414 (3)	0.25149 (13)	0.0218 (4)	
H2BA	0.3292	0.1173	0.2729	0.026*	
H2BB	0.3608	0.0904	0.1860	0.026*	
C3B	0.38592 (9)	0.3623 (3)	0.24999 (13)	0.0230 (4)	
H3BA	0.4014	0.4112	0.3161	0.028*	
H3BB	0.4276	0.3861	0.2248	0.028*	
C4B	0.31721 (9)	0.4781 (3)	0.18995 (12)	0.0228 (4)	
N1A	0.63879 (8)	0.9224 (2)	0.48607 (12)	0.0309 (4)	
H1AA	0.6782	0.9940	0.5136	0.037*	
H1AB	0.5988	0.9745	0.4456	0.037*	
N2A	0.70115 (8)	0.6414 (2)	0.56897 (12)	0.0253 (4)	
H2A	0.7424 (17)	0.704 (4)	0.617 (2)	0.057 (8)*	
N3A	0.68467 (8)	0.4473 (2)	0.57546 (12)	0.0266 (4)	
H3A	0.7227 (16)	0.354 (4)	0.603 (2)	0.051 (8)*	
C1A	0.63984 (9)	0.7296 (3)	0.50616 (12)	0.0230 (4)	
C2A	0.58387 (9)	0.5854 (3)	0.46966 (13)	0.0258 (4)	
H2AA	0.5358	0.6031	0.4241	0.031*	
C3A	0.61415 (10)	0.4134 (3)	0.51456 (14)	0.0272 (4)	
H3AA	0.5897	0.2918	0.5044	0.033*	

### Atomic displacement parameters (Å^2^)

**Table:**

	*U*^11^	*U*^22^	*U*^33^	*U*^12^	*U*^13^	*U*^23^
O1B	0.0265 (7)	0.0171 (7)	0.0430 (8)	0.0006 (5)	−0.0117 (6)	0.0017 (6)
O2B	0.0216 (6)	0.0230 (7)	0.0404 (8)	−0.0020 (5)	−0.0107 (5)	0.0005 (6)
O3B	0.0237 (6)	0.0148 (7)	0.0323 (7)	0.0010 (5)	−0.0057 (5)	−0.0003 (5)
O4B	0.0230 (6)	0.0186 (7)	0.0476 (9)	−0.0014 (5)	−0.0157 (6)	0.0001 (6)
C1B	0.0182 (7)	0.0179 (9)	0.0261 (8)	0.0013 (6)	−0.0019 (6)	0.0017 (6)
C2B	0.0158 (7)	0.0180 (9)	0.0250 (8)	0.0008 (6)	−0.0021 (5)	−0.0006 (6)
C3B	0.0171 (7)	0.0163 (9)	0.0289 (9)	−0.0003 (6)	−0.0019 (6)	0.0020 (6)
C4B	0.0195 (7)	0.0182 (9)	0.0247 (8)	0.0015 (6)	−0.0012 (6)	0.0018 (6)
N1A	0.0221 (7)	0.0196 (9)	0.0387 (9)	−0.0001 (6)	−0.0073 (6)	0.0024 (7)
N2A	0.0186 (7)	0.0175 (8)	0.0306 (8)	0.0012 (5)	−0.0047 (5)	0.0002 (6)
N3A	0.0215 (7)	0.0175 (8)	0.0319 (8)	0.0009 (6)	−0.0037 (5)	0.0007 (6)
C1A	0.0188 (7)	0.0194 (9)	0.0245 (8)	0.0032 (6)	−0.0018 (5)	−0.0012 (6)
C2A	0.0176 (7)	0.0218 (9)	0.0300 (9)	0.0007 (6)	−0.0033 (6)	−0.0021 (7)
C3A	0.0227 (8)	0.0206 (10)	0.0313 (9)	−0.0025 (6)	−0.0011 (6)	−0.0025 (7)

### Geometric parameters (Å, º)

**Table:**

O1B—H1B	0.8200	N1A—H1AA	0.8600
O1B—C1B	1.320 (2)	N1A—H1AB	0.8600
O2B—C1B	1.216 (2)	N1A—C1A	1.339 (2)
O3B—C4B	1.272 (2)	N2A—H2A	0.95 (3)
O4B—C4B	1.252 (2)	N2A—N3A	1.362 (2)
C1B—C2B	1.507 (2)	N2A—C1A	1.346 (2)
C2B—H2BA	0.9700	N3A—H3A	0.94 (3)
C2B—H2BB	0.9700	N3A—C3A	1.340 (2)
C2B—C3B	1.520 (2)	C1A—C2A	1.399 (2)
C3B—H3BA	0.9700	C2A—H2AA	0.9300
C3B—H3BB	0.9700	C2A—C3A	1.367 (3)
C3B—C4B	1.510 (2)	C3A—H3AA	0.9300
			
C1B—O1B—H1B	109.5	H1AA—N1A—H1AB	120.0
O1B—C1B—C2B	117.32 (14)	C1A—N1A—H1AA	120.0
O2B—C1B—O1B	119.68 (15)	C1A—N1A—H1AB	120.0
O2B—C1B—C2B	123.00 (17)	N3A—N2A—H2A	121.7 (17)
C1B—C2B—H2BA	109.0	C1A—N2A—H2A	126.8 (17)
C1B—C2B—H2BB	109.0	C1A—N2A—N3A	108.58 (14)
C1B—C2B—C3B	113.14 (14)	N2A—N3A—H3A	122.1 (17)
H2BA—C2B—H2BB	107.8	C3A—N3A—N2A	108.22 (15)
C3B—C2B—H2BA	109.0	C3A—N3A—H3A	127.1 (17)
C3B—C2B—H2BB	109.0	N1A—C1A—N2A	122.17 (15)
C2B—C3B—H3BA	108.7	N1A—C1A—C2A	130.06 (15)
C2B—C3B—H3BB	108.7	N2A—C1A—C2A	107.76 (16)
H3BA—C3B—H3BB	107.6	C1A—C2A—H2AA	127.0
C4B—C3B—C2B	114.39 (14)	C3A—C2A—C1A	106.08 (15)
C4B—C3B—H3BA	108.7	C3A—C2A—H2AA	127.0
C4B—C3B—H3BB	108.7	N3A—C3A—C2A	109.32 (17)
O3B—C4B—C3B	118.07 (14)	N3A—C3A—H3AA	125.3
O4B—C4B—O3B	122.26 (15)	C2A—C3A—H3AA	125.3
O4B—C4B—C3B	119.66 (16)		
			
O1B—C1B—C2B—C3B	−174.71 (17)	N2A—N3A—C3A—C2A	−1.2 (2)
O2B—C1B—C2B—C3B	5.4 (3)	N2A—C1A—C2A—C3A	1.1 (2)
C1B—C2B—C3B—C4B	−176.32 (15)	N3A—N2A—C1A—N1A	178.99 (17)
C2B—C3B—C4B—O3B	170.20 (17)	N3A—N2A—C1A—C2A	−1.9 (2)
C2B—C3B—C4B—O4B	−10.8 (3)	C1A—N2A—N3A—C3A	1.9 (2)
N1A—C1A—C2A—C3A	−179.8 (2)	C1A—C2A—C3A—N3A	0.0 (2)

### Hydrogen-bond geometry (Å, º)

Cg1 is the centroid of the pyrazolium ring.

**Table:**

*D*—H···*A*	*D*—H	H···*A*	*D*···*A*	*D*—H···*A*
O1*B*—H1*B*···O3*B*^i^	0.82	1.79	2.5832 (18)	164
N1*A*—H1*AA*···O4*B*^ii^	0.86	2.08	2.874 (2)	153
N1*A*—H1*AB*···O2*B*^iii^	0.86	2.07	2.923 (2)	170
N2*A*—H2*A*···O3*B*^ii^	0.95 (3)	1.76 (3)	2.7132 (19)	174 (3)
N3*A*—H3*A*···O4*B*^iv^	0.94 (3)	1.79 (3)	2.672 (2)	156 (3)
C2*A*—H2*AA*···O1*B*^iii^	0.93	2.54	3.372 (2)	148
C3*A*—H3*AA*···O2*B*	0.93	2.47	3.214 (2)	138
C3*B*—H3*BA*···*Cg*1^v^	0.97	2.69	3.511 (2)	142

Symmetry codes: (i) *x*, *y*−1, *z*; (ii) *x*+1/2, −*y*+3/2, *z*+1/2; (iii) *x*, *y*+1, *z*; (iv) *x*+1/2, −*y*+1/2, *z*+1/2; (v) *x*+3/2, *y*+3/2, *z*+1.
